# Genome-Scale Profiling and High-Throughput Analyses Unravel the Genetic Basis of Arsenic Content Variation in Rice

**DOI:** 10.3389/fpls.2022.905842

**Published:** 2022-07-18

**Authors:** Sang-Beom Lee, Gyeong-Jin Kim, Jung-Du Shin, Woojin Chung, Soo-Kwon Park, Geun-Hyoung Choi, Sang-Won Park, Yong-Jin Park

**Affiliations:** ^1^Crop Foundation Research Division, National Institute of Crop Science, Wanju, South Korea; ^2^Residual Agrochemical Assessment Division, National Institute of Agriculture Science, Wanju, South Korea; ^3^Bio-Technology of Multidisciplinary Sciences Co., Wanju, South Korea; ^4^Department of Environmental Energy Engineering, Kyonggi University, Suwon, South Korea; ^5^Reserch Policy Bureau, Rural Development Administration, Wanju, South Korea; ^6^Department of Plant Resources, College of Industrial Sciences, Kongju National University, Yesan, South Korea

**Keywords:** arsenic, ionomics, eQTLs, co-expression network, transcriptome-wide association studies

## Abstract

Ionomics, the study of the composition of mineral nutrients and trace elements in organisms that represent the inorganic component of cells and tissues, has been widely studied to explore to unravel the molecular mechanism regulating the elemental composition of plants. However, the genetic factors of rice subspecies in the interaction between arsenic and functional ions have not yet been explained. Here, the correlation between As and eight essential ions in a rice core collection was analyzed, taking into account growing condition and genetic factors. The results demonstrated that the correlation between As and essential ions was affected by genetic factors and growing condition, but it was confirmed that the genetic factor was slightly larger with the heritability for arsenic content at 53%. In particular, the cluster coefficient of *japonica* (0.428) was larger than that of *indica* (0.414) in the co-expression network analysis for 23 arsenic genes, and it was confirmed that the distance between genes involved in As induction and detoxification of *japonica* was far than that of *indica*. These findings provide evidence that *japonica* populations could accumulate more As than *indica* populations. In addition, the *cis*-eQTLs of AIR2 (arsenic-induced RING finger protein) were isolated through transcriptome-wide association studies, and it was confirmed that AIR2 expression levels of *indica* were lower than those of *japonica*. This was consistent with the functional haplotype results for the genome sequence of AIR2, and finally, eight rice varieties with low AIR2 expression and arsenic content were selected. In addition, As-related QTLs were identified on chromosomes 5 and 6 under flooded and intermittently flooded conditions through genome-scale profiling. Taken together, these results might assist in developing markers and breeding plans to reduce toxic element content and breeding high-quality rice varieties in future.

## Introduction

Ionomics is defined as the study of the composition of mineral nutrients and trace elements in organisms and represents the inorganic components in the cells and tissues of an organism (Salt et al., 2008). Early ionomics focused on the identification or characterization of *Arabidopsis* and yeast mutants (Lahner et al., 2003); however, its application has been extended to high-throughput element profiling to find genes involved in controlling the ionome of an organism (Danku et al., 2013).

Ionomics is a powerful approach to quickly analyzing several samples using the inductively coupled plasma mass spectrometry (ICP-MS). In addition, since transcript and ion profiling can be performed not only on cells regulating plant physiology but also on the whole organism simultaneously, many complex studies on ionomes combined with the genome, transcriptome, proteome, and metabolites have recently been published (Baxter, 2010).

Reportedly, As, a toxic non-essential element, accumulates in rice and other crops due to irrigation with contaminated groundwater, industrialization, mining activity, and use of arsenical pesticides (Duxbury et al., 2003; Liao and Ou, 2005; Williams et al., 2007; Chen et al., 2016). As contamination is not limited to water sources, soil contamination with As induces infertile ears and reduces plant growth, especially in agricultural areas (Kumarathilaka et al., 2018a). Arsenic is a major threat to human health because it is absorbed from the soil and accumulates in edible plant parts (Finnegan and Chen, 2012).

In the environment, As exists in organic and inorganic forms, and the contents of inorganic species, such as arsenate [As(V)] and arsenite [As(III)], are higher in soil than the organic arsenic species, such as monomethylarsonic acid (MMAA) and dimethylarsinic acid (DMAA). The ratio of As(III) and As(V) varies depending on the redox state and redox potential (Eh) of soil; if Eh is negative, As(V) is transformed to As(III), and the proportion of As(III) increases (Yamaguchi et al., 2014). Recent studies based on climate change prediction models have shown that arsenic concentrations in the soil are affected by Co_2_ and temperature. As(III) concentration decreased with increasing CO_2_ concentration, but the toxicity of As(III) to humans increased proportionally with an increase in the temperature compared with that of As(V) (Muehe et al., 2019).

Rice, one of the staple crops consumed by more than 50% of the world's population, is known to be roughly 10 times more likely to assimilate As than other crops (Williams et al., 2007), and therefore, it could be a major source of As toxicity in humans. The mechanism of absorption As(V) is closely related to the phosphate uptake system, whereas As(III) is absorbed by the aquaporins of roots (Ullrich-eberius et al., 1989). It is known that several phosphate transporter genes (e.g., *OsPht1, 1-OsPht1*, and *Pht*) are involved in As(V) absorption, and nodulin 26-like intrinsic proteins (NIPs) have been shown to absorb As(III) (Paszkowski et al., 2002; Bienert et al., 2008; Isayenkov and Matouis, 2008; Ma et al., 2008; Kirk et al., 2009).

The accumulation of ionomes, including arsenic, in plants is regulated by these genes involved in uptake, binding, transport, and sequestration (Baxter, 2009). Therefore, studies to verify the correlation between arsenic and other inorganic components in various populations and to elucidate genetic factors involved in this have been reported, so far (Zhang et al., 2008; Yang et al., 2018). However, the interactions between ionomes are affected not only by genotypes, but also by the environment or the interaction between the environment and the genotypes (Garcia-Oliveira et al., 2009; Hu et al., 2013; Huang and Salt, 2016).

In this study, the interactions between As and other ions were analyzed under non-stress and stress conditions for rice core collections, including *temperate japonica, tropical japonica, indica*, and *aus*, to elucidate the impact of environmental and genotypic differences. In addition, it was attempted to identify the genetic factors regulating As genes through a transcriptome-wide association study on 23 genes known to transport, detoxify, or stress-response (Yang et al., 2012; Nguyen et al., 2014; Most and Papenbrock, 2015; Yamaji et al., 2015; Hwang et al., 2016, 2017; Shi et al., 2016; Das et al., 2017, 2018a,b; Salt, 2017; Latowski et al., 2018; Sun et al., 2018; Wang et al., 2019; Tiwari et al., 2020; Singh et al., 2021).

## Materials and Methods

### Plant Materials

An allele-mining set of 166 accessions was developed using a heuristic algorithm for 4,046 rice accessions collected from 60 countries held by RDA-Genebank (Kim et al., 2007; Zhao et al., 2010). After the rice core set was constructed, association mapping was conducted on eating quality and amylose content, and a rice core collection for the current 430 accessions was established (Lu and Park, 2012a,b; Zhao et al., 2013). A rice core collection was cultivated in non-contaminated and contaminated paddy soil in 2016 and 2017, and then, 273 accessions overlapped by year were selected. The following subspecies of selected 273 accessions were used: 192 temperate *japonica*, 19 tropical *japonica*, 49 *indica*, 8 *aus*, 3 *admixture*, and 2 *aromatic*.

### Field Experiment and Inorganic Component Analysis

Field experiments were conducted on general paddy soil (latitude: 36.670, longitude: 126.855) at Kongju National University and contaminated paddy soil at Hakyeong Mine (latitude: 36.573, longitude: 126.819), Yesan-gun, Chungcheongnam-do, Republic of Korea. The 273 rice accessions were grown in contaminated soil over two years (2016 and 2017), and the ears of each individual were sampled at the yellow ripe stage to analyze accumulation patterns of ionomes in brown rice (Supplementary File 1). In particular, field experiments were conducted under flooded (2016) and intermittently flooded conditions (2017) due to the narrow arable land of the contaminated soil.

The chemical properties of soil were analyzed following the National Academy Aggregation Science (NAAS, 2010). First, soil samples were mixed with distilled water at a 1:5 ratio and kept for 1 h. The pH and EC were then measured (Orion 3 Star, Thermo, USA). To measure cation exchangeability for Ca, Mg, K, and Na in soil, soil samples were mixed with 1 mL of distilled water, 21 mL of HCl, and 7 mL of HNO_3_. Subsequently, the mixture was decomposed using Kjeldahl (C. Gerhardt GmbH & Co., Northants, UK). Afterward, 1 M NH_4_OAc (pH 7.0) was added to the decomposed mixture, mixed by shaking, and filtered using a Whatman No. 42 filter paper (Kang et al., 2018).

After the 273 brown rice accessions were ground using a cyclone miller (PX-MFC 90 D, KINEMATICA, Switzerland), they were decomposed by adding 4 mL of HNO_3_ and 1 mL of distilled water in a microwave oven (UltraWAVE, Milestone, USA). The volume of the decomposed samples was adjusted to 25 mL with distilled water. Micro-elements in plants (As, Zn, Fe, Cu, Mn, Na, and Se) were analyzed using ICP-MS (7700E, Agilent Technologies, USA), and the macro-elements in soil (Ca and Mg) were analyzed using inductively coupled plasma-optical emission spectrometry (Integra XL, GBC, AUS). The R^2^ of the standard calibration curves for each element was 0.999 or more using multi-element standard (Agilent, USA), and it was confirmed that the recovery rates for inorganic components were 80–120% using IRMM-804 rice flour.

Meanwhile, broad-sense heritability for As content was calculated by QTLmax Global (2022) with the following formula:

$H^{2}=\;\frac{\sigma_{g}^{2}}{\sigma_{g}^{2}+\sigma_{e}^{2}}$, where “$\sigma_{g}^{2}$” is the genetic variance, “$\sigma_{e}^{2}$” is the genotype by the annual environmental effect, “σ^2^” is the error variance, and “e” is the annual environmental effect (Le Sech and Christian, 1991).

### DNA Extraction and Whole-Genome Sequencing

DNA and RNA were extracted from the 15-day-old seeds after heading to analyze the SNP and arsenic gene expression levels. Some amino acids or metabolites play important roles in plant growth and development, as well as plant resistance to various stresses (Li et al., 2010). Amino acid metabolism is affected by high night temperature at the early milky stage. As a result, the seed weight and grain quality of rice are determined by changes in gene expression patterns (Liao et al., 2015).

The 15-day-old young seeds were collected and powdered using mortar–pestle. Afterward, genomic DNA was extracted using DNeasy® Plant Mini Kit (QIAGEN, Germany). The concentration of the sample was adjusted to 30 ng μL-1. For next-generation sequencing library preparation, quality control (QC) analysis was performed to ensure that the fragment of the DNA was of the desired size. The extracted DNA samples were quantified using the Quant-iT™ dsDNA High-Sensitivity Assay Kit (Invitrogen, Carlsbad, CA, USA) on an Agilent 2100 Bioanalyzer (Agilent Technologies, Santa Clara, CA, USA). Optical density was measured using Tecan F200 (Tecan, Switzerland), and the quality of the extracted DNA was confirmed by electrophoresis on a 0.7% agarose gel. Short-read sequences were obtained using HiSeq 2500 (Illumina), and next-generation sequencing was performed for genome analysis (Supplementary File 2).

### RNA Sequencing and Transcriptome Analyzes

Total RNA from the samples was extracted using the Total RNA Prep Kit for plant tissues (QIAGEN, Germany). The quality of the extracted RNA was confirmed by electrophoresis on 0.7% agarose gel, and its absorbance was measured using a UV spectrophotometer (UV-2600, SHIMADAZU). The purity and quantification were performed using NanoDrop ND-1000 (Dupont Agricultural Genomics Laboratory). The concentration of the sample was adjusted to 20 ng μL^−1^. The short-read sequence obtained from RNA sequencing was aligned, and the Bowtie (version 1.1.2) and Tophat (version 2.1.0) were used to compare and map with International Rice Genome Sequencing Project 1.0 (Heo et al., 2017; Supplementary File 3).

### Model Selection for GWAS

GWAS is the most powerful statistical tool to analyze the association between traits and SNP markers, and various genetic models are applied to identify quantitative traits. Whole-genome resequencing data were imputed using the Beagle (Browning and Browning, 2007), and then, 3,110,974 SNPs for the rice core collection (273 accessions) were obtained from 808,686 SNPs by adjusting the minor allele frequency (MAF) to be less than 5% and removing the proportion of missing SNPs by 80%. In addition, accurate genome-scale profiling for As was performed by applying the linear mixed model (LMM).

The formula of the LMM is as follows:


(1) $$\begin{array}{r} y=X\beta +Z\text{u}+\varepsilon , \end{array}$$

where “y” is the observed phenotype, “β” indicates the marker information that is a fixed effect, “*u*” indicates the object information that is a random effect, “ε” is the random residual effect, and “X” and “Z” are the associated design metrics. The random effect assumes that *u* is proportional to the normal distribution for the mean and covariates [*u*~N(0, G)] and that ε is proportional to the normal distribution for the means and sum of the error squares [ε~N(0, *I*$\sigma_{\text{ε}}^{2}$)] (Piepho et al., 2008).

On the contrary, in the 2016 and 2017 GWAS results applying GLM, the lambda values were 1.234507 and 1.58868, respectively, which were inflated compared with the LMM (0.781438 and 0.950722) (Supplementary File 4).

Significant QTLs with –log_10_(*p*) > 5 from GWAS results applied with LMM were selected, and linkage disequilibrium was analyzed by calculating allele frequency as r^2^ for each QTL. Candidate genes were identified at the range of ±50 kbp using genome browser of the Rice Annotation Project (https://rapdb.dna.affrc.go.jp/) (Kawahara et al., 2013; Sakai et al., 2013) for QTLs with a low recombination rate (*r*^2^ ≤ 1) (Supplementary File 5). In addition, functional haplotypes for candidate genes were investigated by using genomic sequence information, including the promoter and coding regions. The phenotypic variation for the haplotype group was statistically validated with a one-way ANOVA test.

### Statistical and Network Analyses

Jamovi (version 1.6.12) was used for the correlation analysis between inorganic components. The one-way analysis of variance (ANOVA) was performed for genotypic differences (*japonica, indica*, and *aus*) of the rice core collection. Cytoscape (version 3.7.2) was used for the network analyses between inorganic components based on the Pearson correlation coefficient. However, the network analysis excluded *Admixture* and *Aromatic* collections with small sample numbers (*n* < 5).

## Results

### Variations in the Ionome Among the 273 Rice Accessions

**Figure 1:**
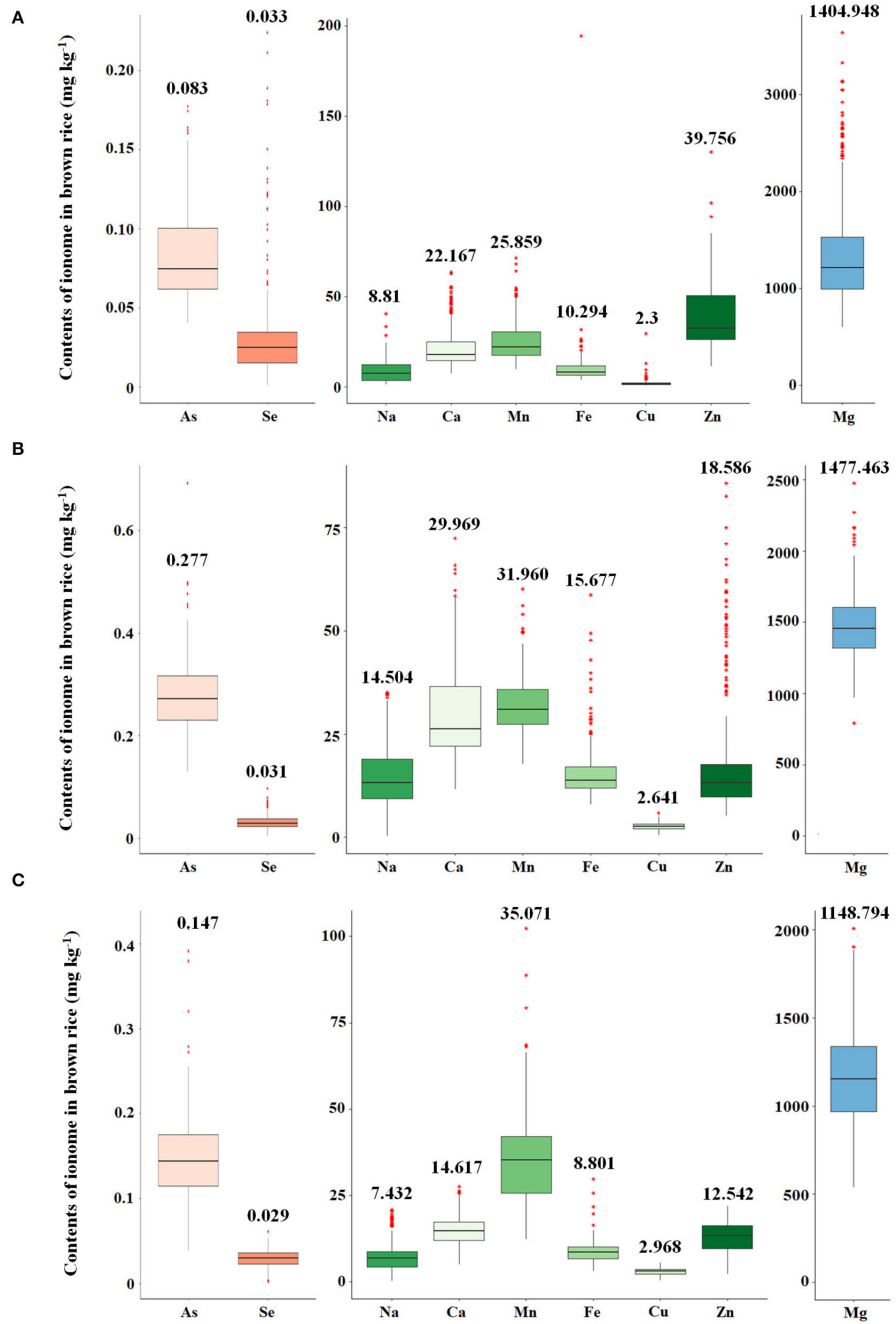
Variation of arsenic and functional ion content for the 273 rice accessions. **(A)** Ion contents in 273 rice grains in non-contaminated soil. **(B)** Ion contents in 273 rice grains under the flooded condition in contaminated soil. **(C)** Ion contents of 273 rice grains under the intermittently flooded condition in contaminated soil.

**Figure 2:**
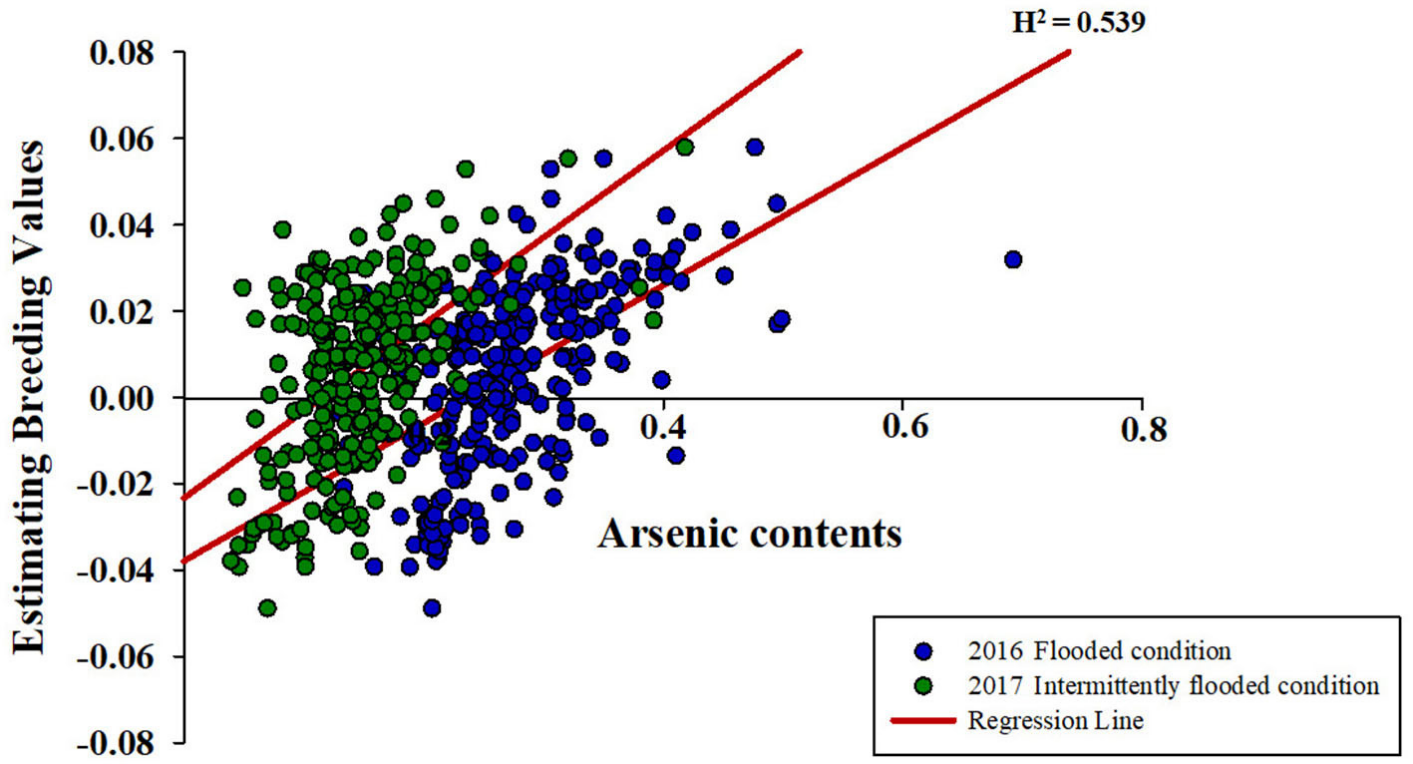
Correlations between estimating breeding values (EBVs) and arsenic contents. Green circle indicates arsenic contents of the 273 rice accessions grown under the flooded condition in contaminated soil. Blue circle indicates arsenic contents of the 273 rice accessions grown under the intermittently flooded condition in contaminated soil.

The variations of As, Se, Na, Ca, Mn, Fe, Cu, Zn, and Mg contents accumulated in brown rice of 273 accessions were analyzed. Under the non-contaminated soil condition, As contents of rice varieties ranged from 0.0407 to 0.1775 mg kg^−1^, with an average content of 0.0833 mg kg^−1^. In 2016, As contents of varieties grown under contaminated soil conditions ranged from 0.1306 to 0.6923 mg kg^−1^, with an average content of 0.2768 mg kg^−1^. This average content increased by approximately 332% compared with the non-contaminated soil condition. The As content of the cultivars in 2017 was grown under the same polluted soil conditions as in 2016, As contents ranged from 0.0391 to 0.4184 mg kg^−1^, with an average of 0.1471 mg kg^−1^, a decrease of approximately 53% compared with the previous year. Functional inorganic components, such as Na, Ca, Mn, Fe, Cu, Zn, and Mg, showed statistically significant differences (*p* < 0.05) in all environmental conditions. It was confirmed that under the contaminated soil condition in 2017, other inorganic components except for Na, Cu, Fe, and Mg increased compared with the contaminated soil condition of the previous year (Figure 1). However, there were no significant differences in Se contents under the contaminated soil conditions in both years. In the 273 rice accessions grown in contaminated soil, the broad-sense heritability (H^2^) of As content in 2016 and 2017 was about 54%, which was found to be affected by both environmental and genetic factors (Figure 2).

### Interaction of Ionomes in Rice Core Collection

**Figure 3:**
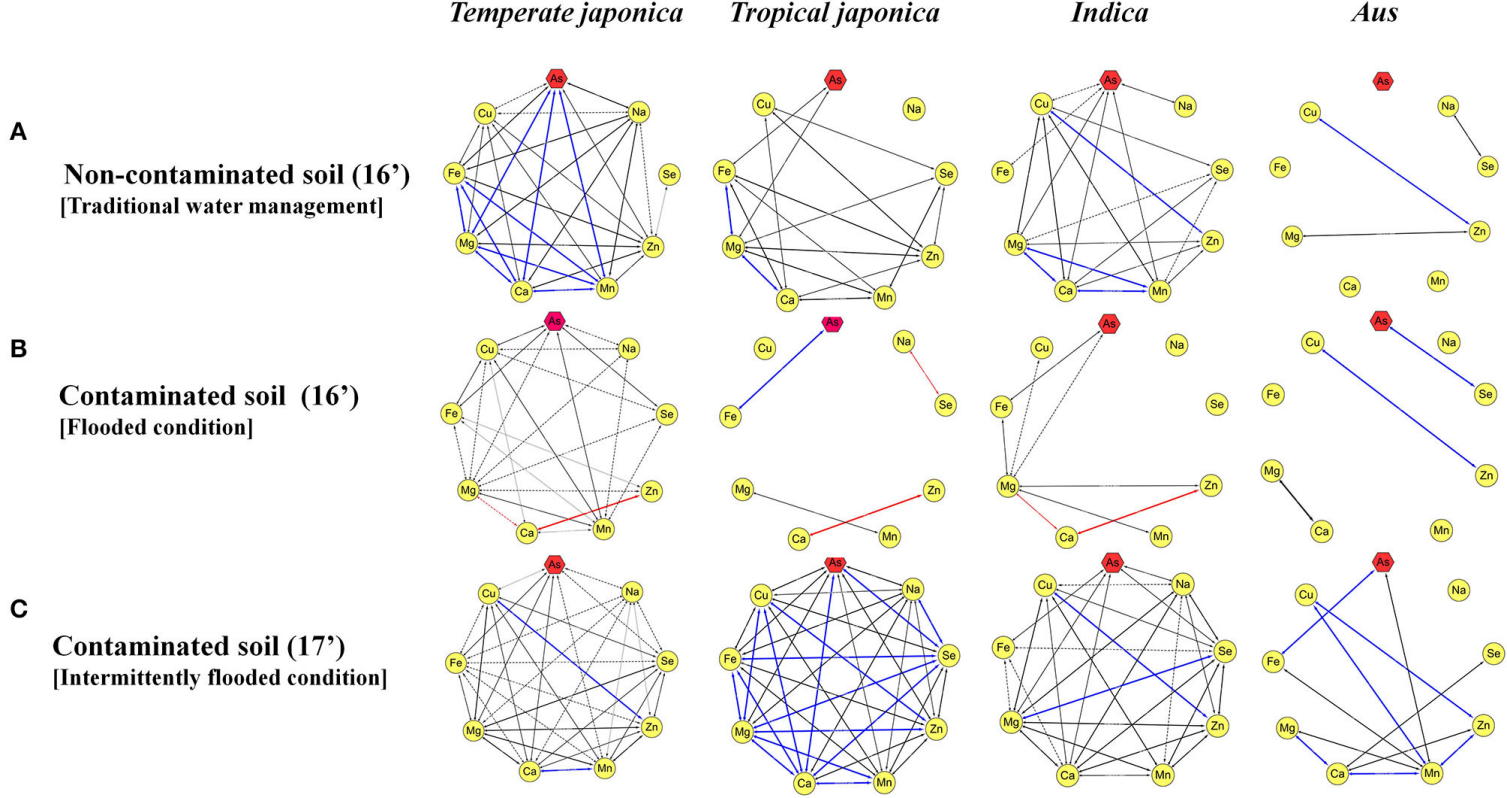
Interaction between arsenic and other inorganic components. The correlation between ionomes was based on the *Pearson coefficient value*. Blue line: positive correlation (0.8 < r <1.0), red line: negative correlation (−1.0 < r < −0.8), black line: positive correlation (0.4 < r <0.8), and dotted line: positive correlation (0.2 < r <0.4). **(A)** The interaction between ionomes of 273 rice grains in non-contaminated soil. **(B)** The interaction between ionomes of 273 rice grains under the flooded condition in contaminated soil. **(C)** The interaction between ionomes of 273 rice grains under the intermittently flooded condition in contaminated soil.

It was confirmed that the accumulation of ionomes in rice is affected by environmental conditions, and the contents of ionomes vary greatly depending on the rice varieties. This is predicted to be due to the genotypic differences due to subspecies, and the ionome content and their interactions were analyzed by classifying them into *temperate japonica, tropical japonica, indica*, and *aus*. Compared with other subspecies, *temperate japonica* exhibited more complex interactions between ionomes under both non-contaminated and contaminated soil conditions (Figures 3A,B). On the contrary, the correlations between ionomes in the rice grown under contaminated soil conditions were increased in all subspecies in the second year (2017) compared with that in the previous year (Figures 3B,C). The direction of correlation between As and other functional minerals varied depending on subspecies and environmental conditions.

### Co-Expression Network Analysis

The subspecies of rice were largely classified into *japonica* and *indica* population; then, the relationship between expression levels and genotypes of 23 genes known to be involved in As transport, detoxification, or As-induced stress response was analyzed for each population (Table 1). The expression networks for 23 As genes were performed using RNA-seq data from the rice accessions grown in unpolluted paddy soils.

**Table 1:** Genes involved in As transport, detoxification, or stress response in rice.

**Gene names**	**Gene symbols**	**Descriptions**
OS02G0745100	LSI1	Aquaporin NIP III subfamily protein, arsenic species (As) uptake, arsenite efflux (Os02t0745100-01)
OS01G0368900	OsGRX4	Glutaredoxin (Grx) family protein, arsenic (As) stress response, drought tolerance (Os01t0368900-01)
OS02G0102300	OsHAC1;1	Arsenate reductase, regulation of arsenic accumulation (Os02t0102300-01)
OS02G0157600	STR8	Arsenate [As(V)] reductase, As(V) tolerance, control of arsenic (As) accumulation (Os02t0157600-01)
OS02G0618100	GRX9	Glutaredoxin (Grx) family protein, arsenic (As) stress response, drought tolerance (Os02t0618100-01)
OS03G0107300	LSI2	Anion transporter, silicon efflux transporter, arsenic species (As) uptake (Os03t0107300-01)
OS03G0108000	ACR2.2	Dual-specificity tyrosine phosphatase CDC25, arsenic metabolism (Os03t0108000-01)
OS03G0195800	OsSultr1;1	Similar to sulfate transporter (fragment) (Os03t0195800-01)
OS03G0747800	OsRCS3	O-Acetylserine(thiol) lyase, cysteine biosynthesis, arsenic detoxification (Os03t0747800-01)
OS04G0249600	OsHAC1;2	Arsenate reductase, sulfurtransferase/rhodanese-like protein, regulation of arsenic accumulation (Os04t0249600-02)
OS04G0620000	OsABCC1	Arsenic (As) detoxification, reduction of As in grains (Os04t0620000-01)
OS05G0497600	OsAIR1	Arsenic-induced RING E3 ligase, abiotic stress response (Os05t0497600-01)
OS05G0554000	MATE2	Arsenic stress response, regulation of plant growth and development, disease resistance (Os05t0554000-03)
OS06G0102300	OsPCS	Phytochelatin synthase 2, cadmium (Cd), and arsenic (As) tolerance (Os06t0102300-03)
OS10G0545700	ACR2.1	Dual-specificity tyrosine phosphatase CDC25, arsenic metabolism (Os10t0545700-04)
OS11G0572500	OsAIR2	Similar to zinc finger, RING type (Os11t0572500-01)
OS02G0232900	OsNIP1;1	A member of the nodulin26-like intrinsic protein (NIP) family, arsenite transporter (Os02t0232900-01)
OS02G0822100	SIET3	Similar to arsenite transport subunit B (Os02t0822100-02)
OS05G0442400	MYB-1	R-R-type MYB-like transcription factor (Os05t0442400-01)
OS08G0152000	NIP 3;2	Nodulin 26-like intrinsic membrane protein, arsenite [As(III)] uptake by lateral roots (Os08t0152000-01)
OS08G0152100	NIP 3;3	A member of the nodulin26-like intrinsic protein (NIP) family, arsenite transporter (Os08t0152100-01)
OS09G0521500	Get3	Similar to arsenical pump-driving ATPase (EC 3.6.3.16) (Os09t0521500-01)
OS01G0955700	OsCLT1	CRT-like transporter, glutathione homeostasis, arsenic tolerance (Os01t0955700-01)

**Figure 4:**
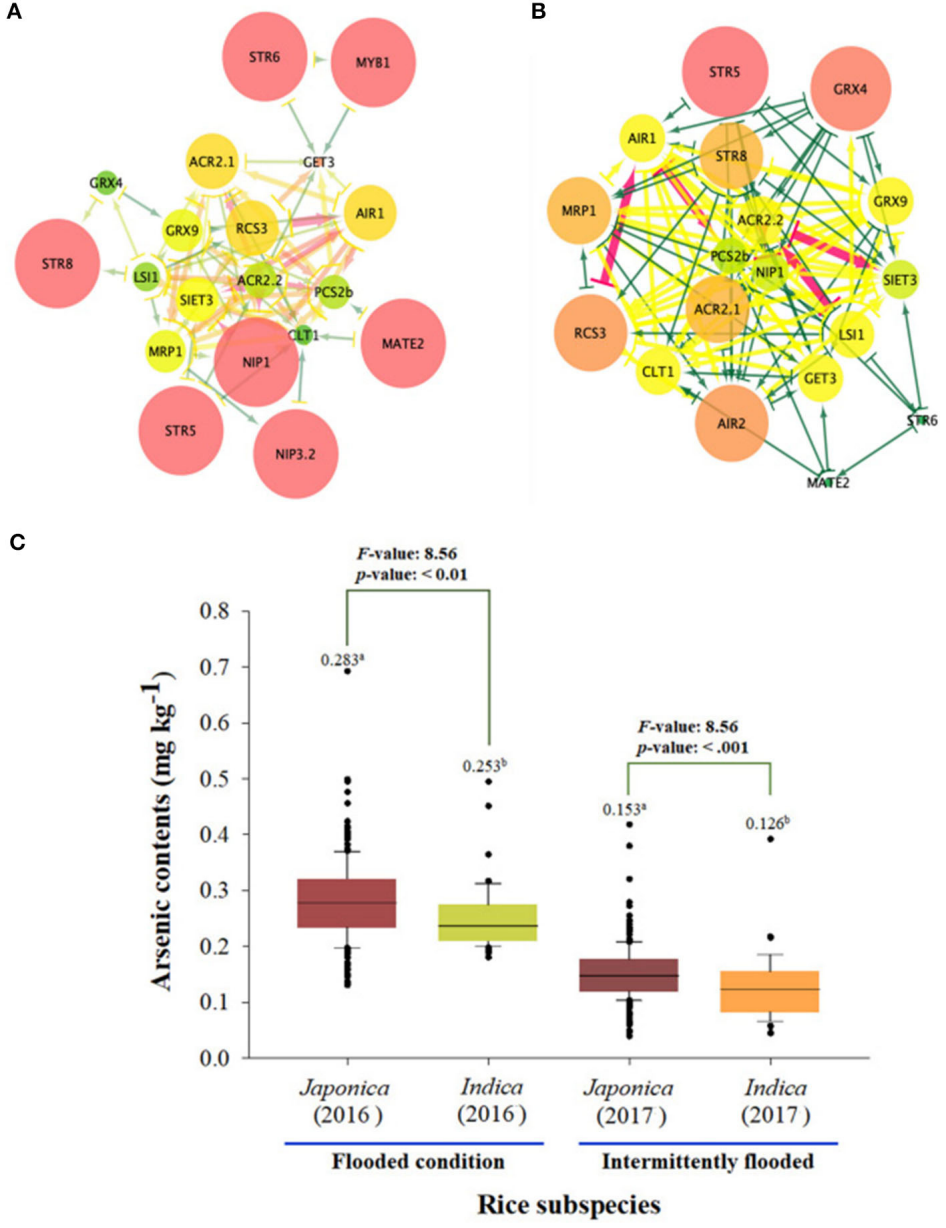
Expression network analysis of As genes between *japonica* and *indica* population. **(A)** The expression network of As-related genes within the *japonica* population. **(B)** The expression network of As-related genes within the *indica* population. **(C)** As content of *japonica* and *indica* under flooded and intermittently flooded conditions. a and b indicates whether there is a statistically significant difference between groups.

The network average clustering coefficient for 23 As genes expression levels was 0.428 in the *japonica* population, which was larger than that in the *indica* population (0.414). That is, it was confirmed that the expression network of the *indica* population was denser than that of *japonica*, so seven essential genes (STR5, STR6, STR8, MYB1, multidrug and toxic compound extrusion 2 [MATE2], NIP1.1, and NIP3.2) were identified in the *japonica* population, and these genes are related to sulfur transferase and As transporter. Seven essential genes (STR5, STR8, GRX4, MRP1, ACR2.1, RCS, and AIR2) involved in sulfur transferase, As translation, and As induction were identified in the *indica* population (Figure 4). As a result of comparing As contents accumulated in grains of rice core collection cultivated in contaminated soil based on network analyses for rice subspecies, the As content of *japonica* under the flooded condition was 0.283 mg kg^−1^, which was significantly higher than that of *indica* (0.253 mg kg^−1^). In addition, the arsenic content of *japonica* was 0.153 even under the intermittently flooded condition, which was significantly higher than that of *indica* (0.126 mg kg^−1^).

### Expression QTL Analyzes

**Figure 5:**
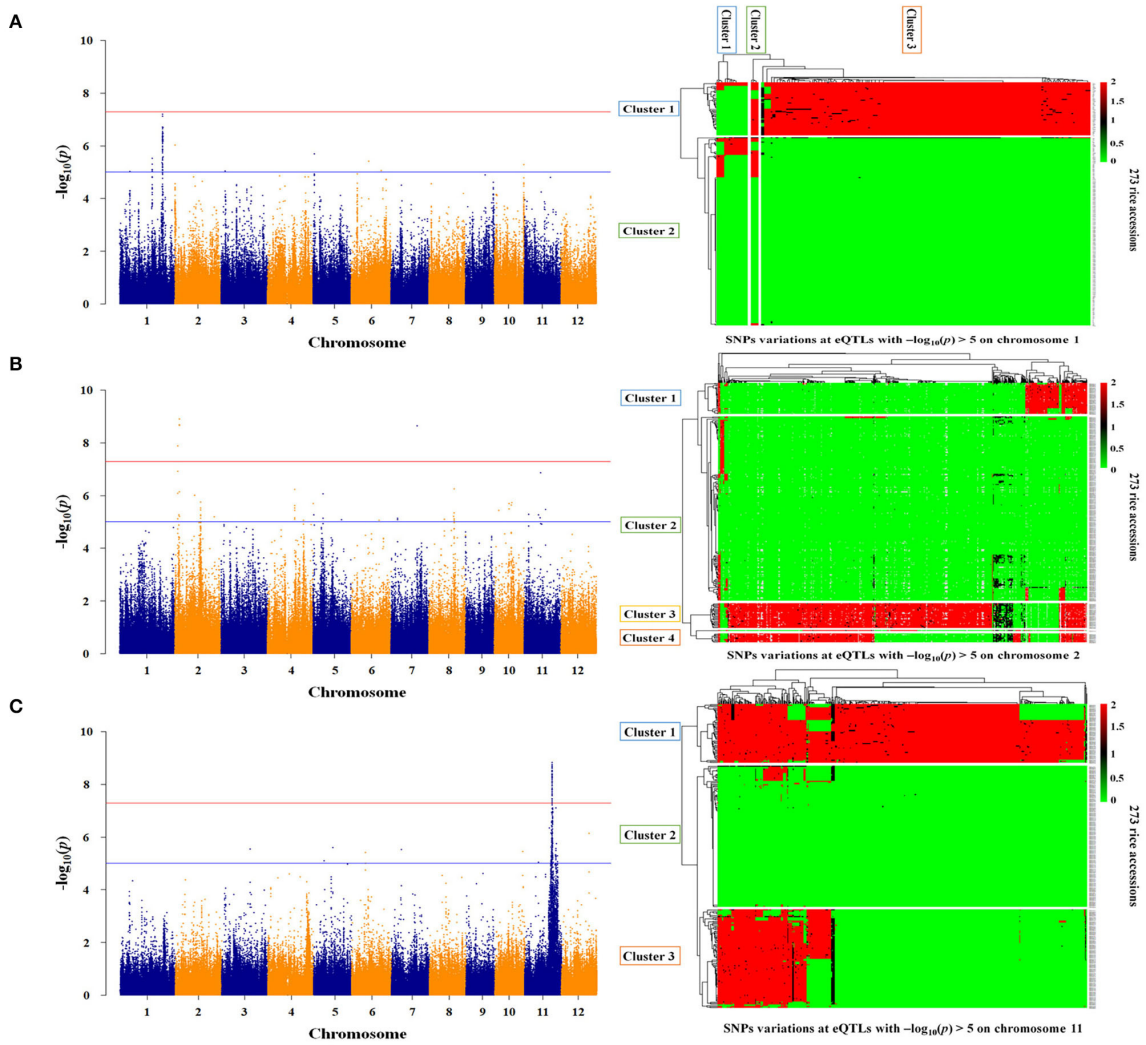
Regulatory factors associated with As gene expression. **(A)** Genome-scale profiling for STR5 expression levels, and SNPs variations at *trans*-eQTLs (25.4–33.6 Mb) in the *japonica and indica* populations. **(B)** Genome-scale profiling for STR8 expression levels and SNPs variations at *cis*-eQTLs (3.0–3.1 Mb and 19.4–20.0 Mb) in the *japonica and indica* populations. **(C)** Genome-scale profiling for AIR2 expression levels and SNPs variations at *cis*-eQTLs (19.2 Mb−25.8 Mb) in the *japonica and indica* populations. The major allele was indicated as 0 (green), the minor allele as 2 (red), and the heterologous allele as 1 (black).

Twelve genes were identified as essential genes in the expression network for As genes. To identify the genetic factors regulating the expression of these genes, the association between the whole genome and the gene expression level was analyzed for the 273 rice accessions. As a result, the associations between expression levels and genotypes for STR5, STR8, and AIR2 were significant as -log_10_(*p*) > 5 (Figure 5). The expression levels of STR5 were associated with *trans*-eQTLs ranging from 25.4 to 33.6 Mb on chromosome 1, and the -log_10_(*p)* for *trans*-eQTLs ranged from 5.03 to 7.21 (Figure 5A). The 18,967 SNPs were identified at *trans*-eQTLs of STR5. These SNPs were clustered into three groups by variations, and 90% *japonica* varieties and 97% *indica* varieties were included in Group 1 and Group 3, respectively. STR5 expression levels of each group were 0.23, 0.35, and 0.30, but there was no statistical significance (Table 2). A total of 121 *trans*-eQTL genes were identified at the *trans*-eQTLs, including 5 at 25 Mb and 31 at 33 Mb. Os01g0635400 (protein-binding) and transcription-regulating Os01g0635550 (ZF-HD homeobox) were detected at 25 Mb, and genes involved in nodulin 20 (Os01g0786500), ABC transporter (Os01g0786000), peroxidase (Os01g0787000), and transcription factor (Os01g0788800) were detected at 33 Mb (Supplementary File 6).

**Table 2:** eQTLs associated with STR5, STR8, and AIR2 expression levels.

**Arsenic** **gene**	**Chromosome**	**Expression quantitative traits loci (eQTLs)**	**Haplotype group**	**Subspecies (number of varieties)**	**Average** **expression levels**	**Differences in gene expression between haplotype groups**
STR5	1	*trans*-eQTLs 25.4–33.6 Mb	Group 1	*japonica* (160)	0.23^a^	
				*indica* (0)		
				Others (0)		
			Group 2	*japonica* (17)	0.35^a^	*F*-value 2.06 *p*-value 0.138
				*indica* (1)		
				Others (3)		
			Group 3	*japonica* (0)	0.30^a^	
				*indica* (42)		
				Others (10)		
STR8	2	*cis-eQTLs* 3.0–3.1 Mb	Group 1	*japonica* (177)	0.22^a^	
				*indica* (27)		
				Others (3)		
			Group 2	*japonica* (0)	0.33^a^	*F*-value 1.63 *p*-value 0.220
				*indica* (5)		
				Others (0)		
			Group 3	*japonica* (0)	0.21^a^	
				*indica* (11)		
				Others (10)		
AIR2	11	*cis-eQTLs* 19.2–25.8 Mb	Group1	*japonica* (132)	1.89^a^	
				*indica* (0)		
				Others (3)		
			Group 2	*japonica* (41)	1.85^a^	*F*-value 13.5 *p* < 0.001
				*indica* (0)		
				Others (0)		
			Group 3	*japonica* (4)	1.67^b^	
				*indica* (43)		
				Others (10)		

*a and b indicates whether there is a statistically significant difference between groups*.

The expression levels of STR8 and AIR2 showed high association at *cis*-eQTLs on chromosome 2 and chromosome 11, respectively. The *cis*-eQTLs for STR8 were identified at 3.0 to 3.1 Mb and 19.4 to 20.0 Mb on chromosome 2. The –log_10_(*p)* for *cis*-eQTLs ranged from 5.1 to 8.9 (Figure 5B). The 226 SNPs at 3 Mb were identified and clustered into three groups by SNPs variations. All *japonica* and 63% *indica* varieties were included in Group 1, and 26% *indica* varieties were included in Group 3. The expression levels of STR8 in each group were 0.22, 0.33, and 0.21, but there was no significant difference between groups. The 376 SNPs were identified and clustered into two groups at *cis*-eQTLs within 19.4–20 Mb. All *japonica* cultivars and 72% *indica* varieties were included in Groups 1 and 2, respectively, but there was no association between STR8 expression and SNPs variations at *cis*-eQTLs (Table 2). The 20 *cis*-eQTL genes were detected at *cis*-eQTLs of STR8. The *cis*-eQTLs genes involved in leucine-rich repeat domain (e.g., Os02g0156400) and arsenate reductase (Os02g0157600) were identified at 3 Mb. In addition, five cis-eQTLs genes, including chloroplast development protein (Os02g0539600), were identified at 19.2–20 Mb (Supplementary File 6).

The 15,388 SNPs were identified at 19.2 Mb−25.8 Mb associated with AIR2 expression, and -log_10_(*p)* for *cis*-eQTLs ranged from 5 to 8.84 (Figure 5C). The *cis*-eQTLs were clustered into three groups, and 75% *japonica* and all *indica* varieties were included in Groups 1 and 2, respectively. AIR2 expression levels were 1.89, 1.85, and 1.67 in each group, and there was a significant difference (*p* < 0.001) in Group 1 and Group 3 (Table 2). The 591 *cis*-eQTL genes for AIR2 were detected, and it was confirmed that a number of genes are related to leucine-rich repeat domains (e.g., Os11g0568800), serine/threonine kinase activity (Os11g0569300), heat shock (Os11g0578500), and RNA binding (Os11g0579900). In addition, the AIR2 was detected as a *cis*-eQTLs gene (Supplementary File 6).

### QTLs Identification for As Content Through GWAS

**Figure 6:**
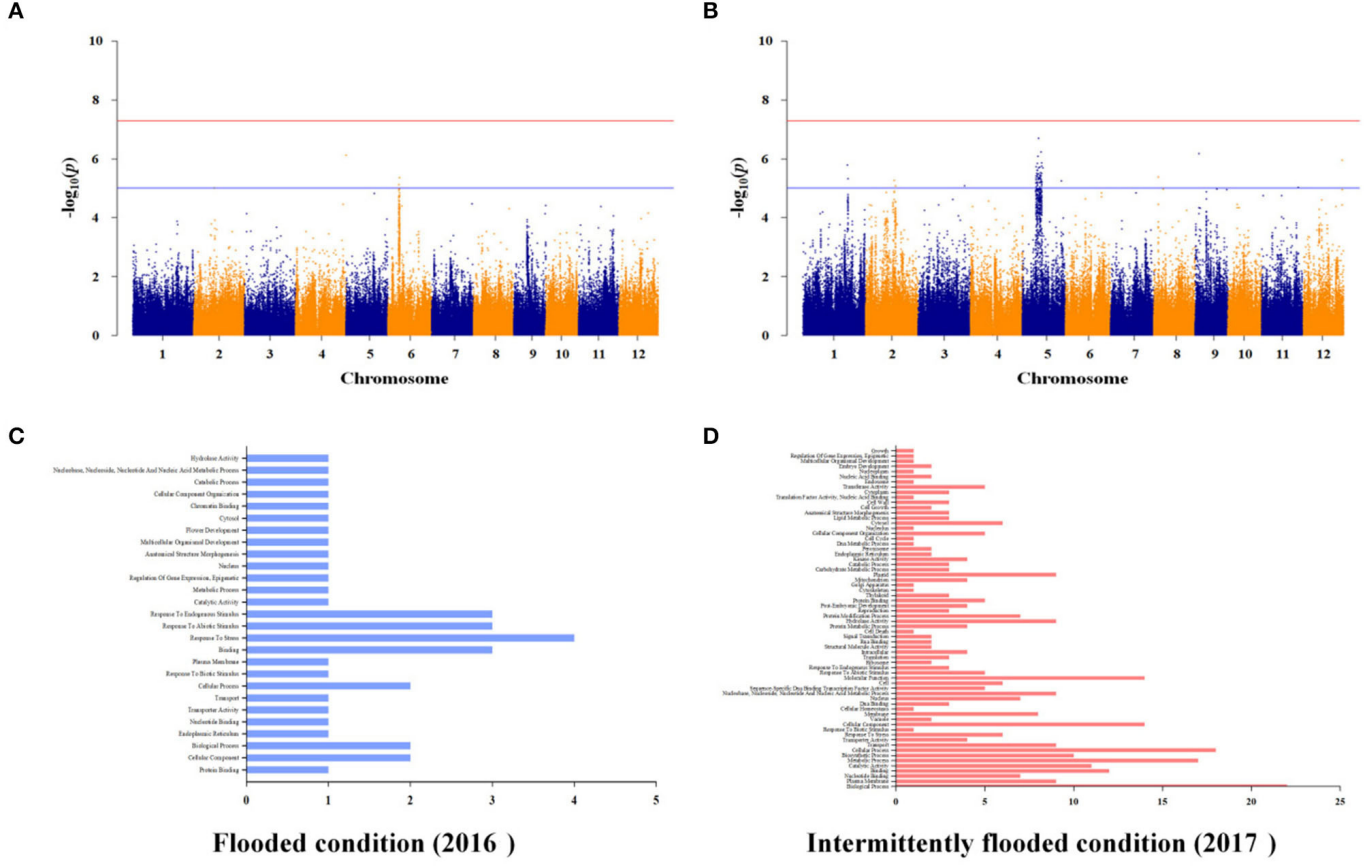
Whole genome-scale profiling for As under flooded and intermittently flooded conditions in contaminated soil. **(A)** Significant SNPs with -log_10_(*p*) > 5 were identified on chromosome 6 under the flooded condition. **(B)** Significant SNPs with -log_10_(*p*) > 5 identified on chromosomes 1 and 5 under the intermittently flooded condition. **(C)** Ten candidate genes under the flooded condition were identified within strongly linked SNPs (*R*^2^ ≤ 1), and a number of candidate genes were involved in abiotic stress response. **(D)** The 150 candidate genes under the intermittently flooded condition were identified within strongly linked SNPs (*R*^2^ ≤ 1), and a number of candidate genes were involved in biosynthetic process, membrane, and plastid.

To identify additional As-related genes other than known major genes and to investigate genetic factors associated with arsenic in plants by varying growing conditions under the same stress environment, we performed GWAS of the rice core collections, grown under contaminated soil conditions in 2016 and 2017. The contaminated soil was maintained under the flooded condition in 2016 and was maintained under the intermittently flooded condition in 2017. Under the flooded condition, As-associated QTLs (As-QTLs) were identified at 8.0 and 8.32 Mb on chromosome 6 and *p*-values were significant as 5.12 and 5.36, respectively (Figure 6A). As a result of LD (linkage disequilibrium) for As-QTLs, it was confirmed that the linkage equilibrium was weakly linked (R^2^ <0.5) within 8.32 Mb and strongly linked (R^2^ ≤ 1) between arsenic-related SNPs within 8.0 Mb (Supplementary File 5). Ten candidate genes were identified within ±50 kb based on 8.0 Mb, and a number of candidate genes were identified that respond to abiotic stress (Figure 6C). In particular, Os06g0254200 for ion channel activity and potassium transport, Os06g0254300 for calcium ion binding, and Os06g0255100, an iron-dependent oxygenase protein, were detected as As-related candidate genes (Supplementary File 7).

Under the intermittently flooded condition, As-QTLs were identified from 30.7 to 30.8 Mb and 9.6 to 14.2 Mb and 27.1 Mb on chromosomes 1 and 5, respectively (Figure 6B). The *p*-values of As-QTLs ranged from 5 to 6.7, and linkage equilibrium was strongly linked with *R*^2^ ≤ 1 (Supplementary File 5). The 150 candidate genes on chromosomes 1 and 5 were identified within ±50 kb based on each As-QTL. Candidate genes involved in biosynthetic process, membrane, and plastid were detected under the intermittently flooded condition, unlike candidate genes identified under the flooded condition (Figure 6D). In addition, Os01g0733001 (NRAMP3), which transports metal ions, and Os05g0279400 (RFP), which regulates innate immunity and disease resistance, were detected at As-QTLs (Supplementary File 7). GWAS results for other functional ions are not presented in the main text and are given in Supplementary File 8.

### Selection of Low-Arsenic Rice Varieties

AIR2 showed a significant difference in expression between *japonica* and *indica* at the *cis*-eQTL (*p* < 0.001), so it was verified whether the arsenic content differs by rice subspecies in genomic sequences of AIR2. The haplotype group for AIR2 was classified into three groups, and SNP variations between groups were confirmed in nonsynonymous SNPs (Ala^134^ → Thr^134^, Gly^130^ → Ser^130^, Ile^101^ → Thr^101^). The 104 *japonica* varieties belonged to Hap1, and the average As content under flooded and intermittently flooded conditions was 0.295 and 0.161 mg kg^−1^, respectively. Hap2 included 54 *japonica* and 18 *indica* varieties, and the average As content under flooded and intermittently flooded conditions was 0.261 and 0.143 mg kg^−1^, respectively. Hap3 included 19 *japonica* and 25 *indica* varieties, and the average As content under flooded and intermittently flooded conditions was 0.251 and 0.134 mg kg^−1^, respectively, which were significantly lower (*p* < 0.01) than those of Hap1 and Hap2.

**Figure 7:**
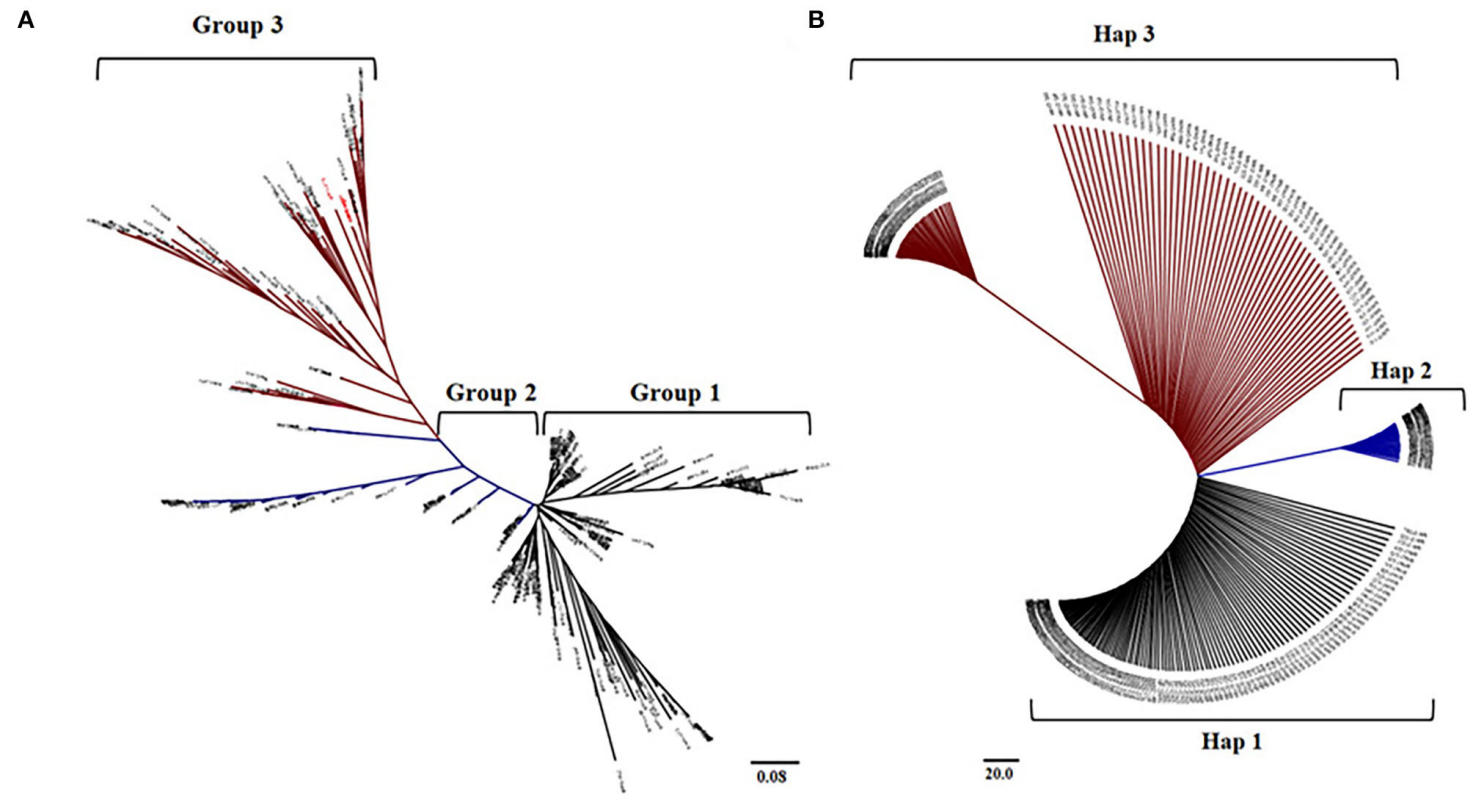
Comparison between haplotype group and cis-eQTLs of AIR2. **(A)** Neighbor-joining tree and clustering groups for 273 accessions at the cis-eQTLs associated with AIR2 expression levels. **(B)** Neighboring binding trees and haplotype groups of 273 accessions to the genomic sequence of AIR2.

The haplotype groups for AIR2 were similar to the groups clustered in the *cis*-eQTLs of AIR2. Based on the results of these analyses, rice varieties with low expression levels and low As content were selected from *japonica* and *indica*, respectively (Figure 7).

## Discussion

### Variation and Interaction of Ionomes in Rice Core Collection

Partitioning the core collection based on rice subspecies and estimation of the interaction between As and other functional minerals demonstrated that environmental and genetic factors contribute to these interactions. The ionome accumulation of 273 rice grains cultivated in contaminated paddy soil was generally increased compared with unpolluted paddy soil conditions in the same year. Na, Mn, Fe, Cu, and Mg including arsenic increased, while Se, Ca, and Zn decreased in the contaminated soil (flooded condition). Nicula et al. (2013) investigated whether the properties of metals and the particularities of each species affect the uptake capacity in *Phaseolus vulgaris* and *Zea mays* and found that Fe, Cu, and Zn content of the two species was increased in contaminated soil compared with unpolluted soil. In addition, Williams et al. (2009) confirmed that Zn and Se significantly decreased in rice as the arsenic concentration increased. The results of these previous studies are consistent with the variations in ionome contents for the 273 rice accessions.

The correlations between ionomes were decreased in contaminated soil (flooded condition) compared with unpolluted soil, and it was confirmed that there was a difference by rice subspecies. The findings also revealed that the correlations between ionomes under the intermittently flooded condition were increased compared with those under the flooded condition. Therefore, it can be inferred that the contents and interaction between As and functional ions vary depending on the rice subspecies and water management. Although the genetic basis for the correlation between trace elements or toxic elements has not yet been established (Tan et al., 2020), studies have demonstrated the influence of subspecies on As, Hg, Pb, and Cd contents in *japonica* and *indica* rice (Meharg et al., 2009; Jiang et al., 2012; Norton et al., 2012). In addition, Wang et al. (2020) reported that the variation in accumulation and translocation of As among 74 main rice cultivars, including 66 *japonica* and 8 *indica* cultivars, was influenced by Si, P, Fe, and Mn contents.

Water management is an effective way to reduce the absorption of toxic ions, such as As, in crops. Rahaman and Ashim (2013) reported that arsenic content by approximately 24% is reduced and Fe and Zn are also reduced to lower levels under the intermittently flooded condition. Although there are environmental factors for the annual As content of 273 rice varieties, it is consistent with previous studies in that As content was decreased by 46.8% under the intermittently flooded condition compared with the flooded condition, and Na, Fe, and Zn also were decreased by 43.8 and 32.5%, respectively. However, the intermittently flooded condition induces oxidative stress and facilitates Cd accumulation, particularly in crops, so the rice core collections were classified into subspecies, and eQTL analyses were performed on 23 genes known as As induction or resistance to identify the genetic factors.

### Co-Expression of As-Related Genes

STR5, STR6, STR8, MYB1, MATE, NIP1.1, and NIP3.2 were identified as essential proteins in the expression networks of 23 As genes of the *japonica* population, and the clustering coefficient of these genes was 0.5, which was larger than that of other genes. The clustering coefficient of GET3 was the smallest at 0.23. STR5, STR8, GRX4, MRP1, ACR2.1, RCS, and AIR2 were identified as essential genes with clustering coefficients ranging from 0.45 to 0.5 in the expression network of the *indica* population. The clustering coefficients of STR6 and MATE2 were the smallest at 0.23. The average clustering coefficient of *japonica* population was 4.28, which was larger than that of *indica* population, but the shortest paths accounted for 30% of the total path, which was less than that of *indica*. Here, the clustering coefficient describes the relatedness of two proteins and measures the importance of edges in a protein–protein interaction network, thus facilitating identification of key proteins in populations (Wang et al., 2012). That is, although many essential arsenic genes are present in the *japonica* population, the distance between As-induced and As-resistant genes is longer than that of *indica*, so it is likely to be easily exposed to the risk of As. This hypothesis was indirectly verified through the results of the As content of *japonica* and *indica* in the rice core collections grown in contaminated soil. On the contrary, the expression levels of STR5, STR8, and AIR2 among the 14 essential genes were -log_10_(p) > 5, which had high associations with the SNPs of core collections. Reportedly, sulfur assimilation and GSH are upregulated in response to oxidative stress in plants exposed to heavy metals (Na and Salt, 2011). Sulfur assimilation plays an important role in minimizing As absorption and transfers to crops and arsenic detoxification (Dixit et al., 2015). *STR5, STR6*, and *STR8* are genes associated with sulfur assimilation and are known to regulate the As accumulation (Most and Papenbrock, 2015).

AIR2 (As-induced RING E3 ligase 2) encodes RING E3 ubiquitin ligase. Heterogeneous overexpression of OsAIR2 has been reported to positively regulate a plant growth in Arabidopsis or rice in response to As(V) stress. In addition, OsKAT1 (3-ketoacyl-CoA thiolase protein), the physical interaction partner of OsAIR2, is degraded and ubiquitinated by OsAIR2 through the 26S proteasome degradation pathway (Lim et al., 2014; Hwang et al., 2016, 2017). The eQTLs of STR5 and STR8 were not directly related to the gene expression, but the expression level of AIR2 showed a significant difference between groups at the *cis*-eQTLs. In particular, these *cis*-eQTLs include AIR2 genomic sequences, and homogeneous and heterogeneous SNPs were observed in Groups 1 (75% *japonica*) and 3 (100% *indica*), respectively. These findings suggest that SNP variations at the *cis*-eQTL associated with OsAIR2 regulate resistance to As stress, and it can be inferred that *indica* is less likely to accumulate As than *japonica*.

A number of *trans* and *cis*-eQTL genes were identified, such as nodulin 20, ABC transporters, leucine-rich repeat domains, and transcription factors at the eQTLs associated with the expression of STR5, STR8, and AIR2. Nodulin 20 (EN20) is a signaling mediator that activates plant defenses under stress, and OsABCC1, a type C ATP-binding cassette transporter (OsABCC) family, is known to detoxify As in grains (Wu et al., 2011; Song et al., 2014). In addition, the leucine-rich repeat domain (LRR) is involved in negative regulator-programmed cell death, tolerance to oxidative stress, and salt stress, but the biological function of most of the LRR gene has not been clearly elucidated in the plant genome (De Lorenzo et al., 2009; Oh et al., 2010; Pitorre et al., 2010; Hwang et al., 2011).

### Identification of QTLs and Candidate Genes for as Content

In general, GWAS is the most powerful statistical method that provides a genetic basis for complex traits and has been applied to major agricultural traits, including toxic minerals, yield, flowering time, and disease resistance, among others, in rice (Huang et al., 2010, 2012; Famoso et al., 2011; McCouch et al., 2016). In rice, the alleles with large effects have been retained and fixed by evolution, human selection, and inbreeding, and therefore, GWAS is widely used to investigate these complex alleles in rice to uncover the genetic basis and effects associated with various traits.

In this study, GWAS was performed by applying LMM to arsenic content of the rice core collection under different water management conditions in the contaminated soil to uncover the genetic factors associated with As. GWAS results applied with LMM were verified by comparison with a general linear model (GLM). A lambda value ranging from 0 to 1 is a measure of asymmetric association indicating the strength of the relationship between the independent and dependent variables and is used to validate the *p*-value from the GWAS results (Hartwig, 1973; Kim et al., 2019).

As-associated SNPs were mapped on chromosomes 6 and 5 under flooded conditions and intermittently flooded conditions, respectively. The association of arsenic with the genome in crops may vary depending on growing conditions. Norton et al. (2019) reported various QTLs associated with arsenic through annual comparisons by water management systems. Moreover, it is known that microorganisms directly or indirectly affect the mobility and biological availability of arsenic in rice paddies, depending on the water management system; then, arsenic uptake and translocation can be decreased or increased by altered expression of transporter genes in plants (Kumarathilaka et al., 2018b). Concordant with the findings of previous studies, this study confirmed that As-related QTLs showed different patterns under water management conditions and that there were several candidate genes involved in physiological responses under intermittently flooded conditions. In a follow-up study, it is necessary not only to verify these candidate genes based on the GWAS results for As, but also to further prove the genetic relationship between arsenic and functional ions by exploring SNPs co-localized with other functional ions.

### Selection of Low-Arsenic Rice Varieties

The 273 rice accessions were classified according to rice subspecies in genomic sequences and *cis*-eQTLs of AIR2. The groups clustered in *cis*-eQTLs mostly coincided with the haplotype groups of AIR2, and *indica* varieties had lower AIR2 expression and As content compared with *japonica*. These results suggest that *indica* is relatively less likely to be exposed to As risk compared with *japonica* and also means that low expression of AIR2 can reduce As accumulation in rice. Therefore, rice varieties (eight indica, two temperate *japonica*, and two tropical *japonica*) with low AIR2 expression and low As content were finally selected based on these results.

## Conclusion

The correlations between arsenic and functional ions were affected by rice subspecies and growing condition through the water management. As was reduced under the intermittently flooded condition compared with the flooded condition, but As reduction through the water management has limitations in terms of the rice-growing condition. Therefore, it was verified that the arsenic accumulation was lower in *indica* than in *japonica* through the association studies between transcriptome and genome data of rice core collections. AIR2 (arsenic-induced RING finger protein) expression for *indica* was relatively lower than that of *japonica* in its *cis*-eQTLs, and it was confirmed that the arsenic content of the group including a number of *indica* was also low in haplotype group for AIR2. Finally, rice varieties with low AIR2 expression and low arsenic content were selected. Therefore, the selected rice varieties are valuable as breeding materials, so verification through follow-up studies is required. In addition, it is necessary to develop a low-arsenic marker that can be applied to all rice varieties by converting three nonsynonymous SNPs into KASP markers in future through the SNP mutation information of AIR2.

## Data Availability Statement

The whole genome resequencing information for the rice core collection (273 rice accessions) is available in “Rice evolution analysis based on the chloroplast genome” by NCBI (https://www.ncbi.nlm.nih.gov/).

## Author Contributions

S-BL and S-WP designed the research. S-BL performed the experiments and wrote the initial manuscript. J-DS provided technical advice. G-JK and G-HC analyzed arsenic contents of rice core collection. S-WP and Y-JP supervised the study. Y-JP preprocessed rice whole-genome resequencing information and whole transcriptome data used in this study. S-KP, J-DS, and WC reviewed the manuscript. All authors contributed to the article and approved the submitted version.

## Funding

This study was funded by the Next-Biogreen 21 Programme, under grant agreement no. PJ013405, Studies on the metabolic behavior of harmful substances, such as soil persistence pesticides and crop uptake no. PJ01594403, and Development of low-allergy soybean breeding materials and molecular marker to improve added value no. PJ01678402.

## Conflict of Interest

J-DS was employed by Bio-Technology of Multidisciplinary Sciences Co. The remaining authors declare that the research was conducted in the absence of any commercial or financial relationships that could be construed as a potential conflict of interest.

## Publisher's Note

All claims expressed in this article are solely those of the authors and do not necessarily represent those of their affiliated organizations, or those of the publisher, the editors and the reviewers. Any product that may be evaluated in this article, or claim that may be made by its manufacturer, is not guaranteed or endorsed by the publisher.

## Supplementary Material

Supplementary File 1 Regional climate information for 2016 and 2017 in Yesan-gun, Chungcheongnam-do, Republic of Korea.

Supplementary File 2 DNA sequencing and rice core collection information for 279 accessions.

Supplementary File 3 Expression levels (FPKM) of arsenic genes for 234 rice accessions.

Supplementary File 4 Differences between genetic models applied to GWAS for arsenic content in brown rice. It was verified that the *p*-value of the GWAS result was inflated using the lambda values (a value between 0 and 1). **(A)** GWAS results of applying the general linear model (GLM) and the linear mixed model (LMM) to the arsenic content under the flooded condition. **(B)** GWAS results of applying the general linear model (GLM) and the linear mixed model (LMM) to the arsenic content under the intermittently flooded condition.

Supplementary File 5 Linkage disequilibrium for As-related QTLs. **(A,B)** Linkage disequilibrium for arsenic-associated QTLs on chromosome 6 under the flooded condition. **(C–E)** Linkage disequilibrium for arsenic-associated QTLs on chromosome 5 under the intermittently flooded condition. **(F,G)** Linkage disequilibrium for arsenic-associated QTLs on chromosome 1 under the intermittently flooded condition.

Supplementary File 6 eQTLs candidate genes associated with STR5, STR8, and AIR2 expression levels.

Supplementary File 7 Candidate genes identified from GWAS for arsenic contents under flooded and intermittently flooded conditions.

Supplementary File 8 Genome-wide scale profiling for functional ions. **(A)** QTLs associated with functional ions in brown rice under the flooded condition. **(B)** QTLs associated with functional ions in brown rice under the intermittently flooded condition.
