# Supplementary figures and images for: Genome-Scale Profiling and High-Throughput Analyses Unravel the Genetic Basis of Arsenic Content Variation in Rice
Source: Front Plant Sci. 2022 Jul 18;13:905842. doi: 10.3389/fpls.2022.905842 (PMC9361212; doi:10.3389/fpls.2022.905842)

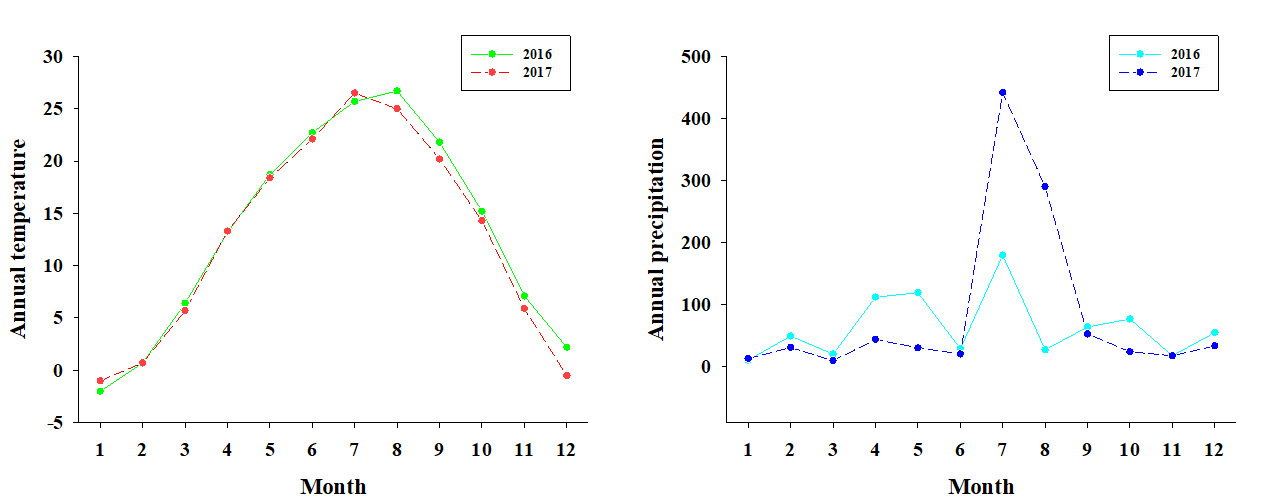

Supplement: Supplementary File 5 — Linkage disequilibrium for As-related QTLs. (A,B) Linkage disequilibrium for arsenic-associated QTLs on chromosome 6 under the flooded condition. (C–E) Linkage disequilibrium for arsenic-associated QTLs on chromosome 5 under the intermittently flooded condition. (F,G) Linkage disequilibrium for arsenic-associated QTLs on chromosome 1 under the intermittently flooded condition. [file Image_1.jpg]

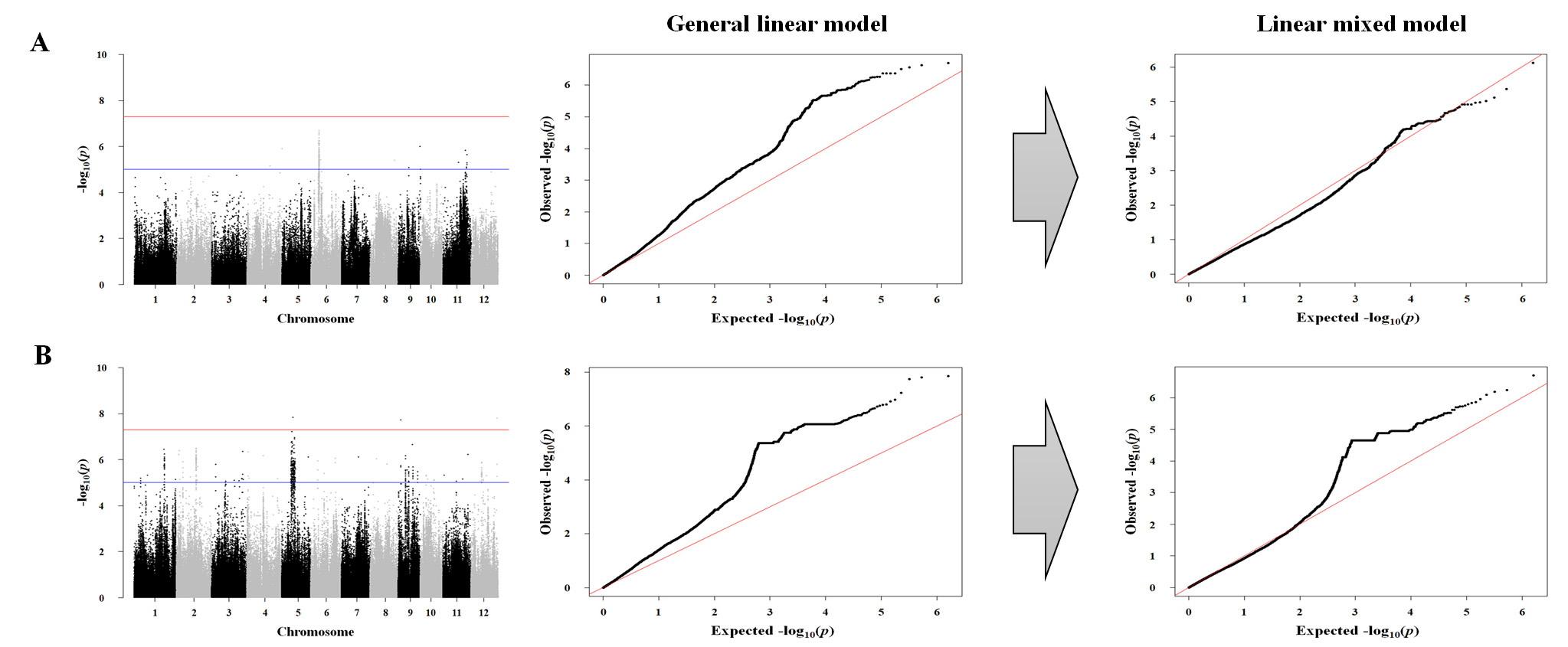

Supplement: Supplementary File 6 — eQTLs candidate genes associated with STR5, STR8, and AIR2 expression levels. [file Image_2.JPEG]

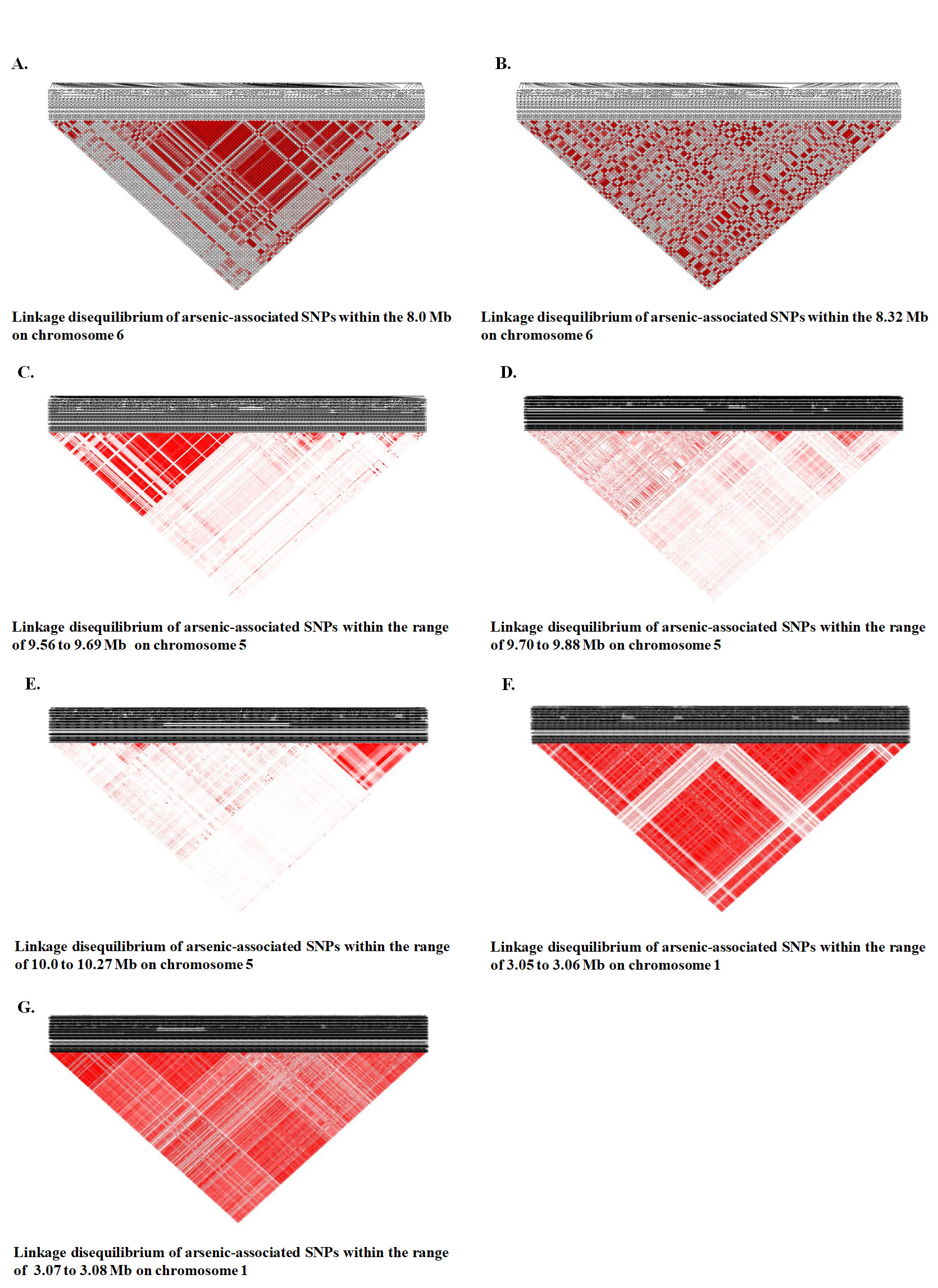

Supplement: Supplementary File 7 — Candidate genes identified from GWAS for arsenic contents under flooded and intermittently flooded conditions. [file Image_3.JPEG]

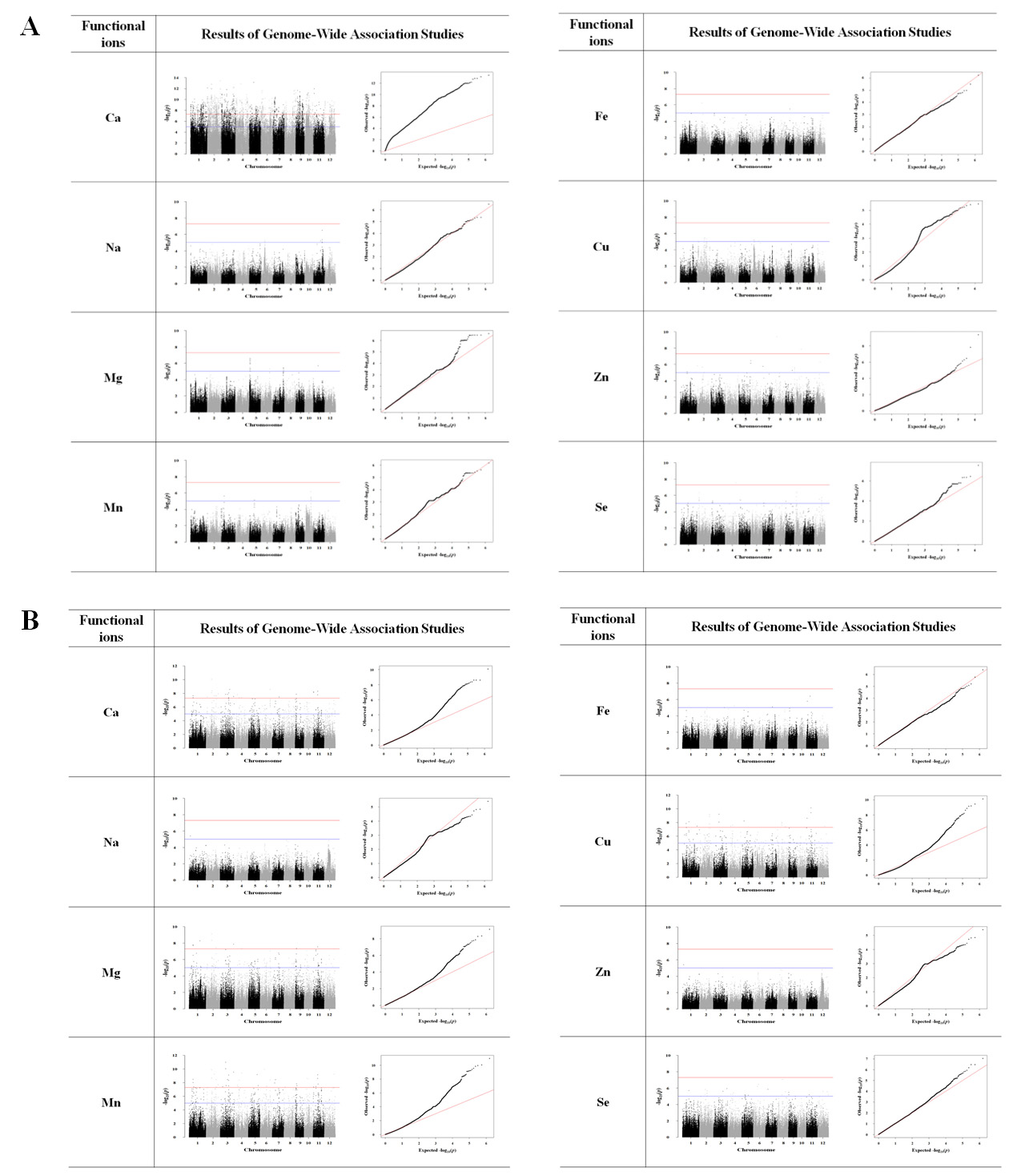

Supplement: Supplementary File 8 — Genome-wide scale profiling for functional ions. (A) QTLs associated with functional ions in brown rice under the flooded condition. (B) QTLs associated with functional ions in brown rice under the intermittently flooded condition. [file Image_4.JPEG]
